# Genomes Offer Ecological Clues to Viruses That Target Ubiquitous Ocean Bacteria

**DOI:** 10.1371/journal.pbio.0030184

**Published:** 2005-04-19

Cyanobacteria have a long and checkered past. When their ancestors first appeared some 3 billion years ago, earth's atmosphere likely contained mostly carbon dioxide, along with hydrogen sulfide, ammonia, nitrogen, and water vapor. Thought to be the first photosynthesizers, cyanobacteria forebears used water from their ocean habitat, carbon dioxide, and sunlight to make sugar, and produced oxygen as waste—the kiss of death for most ancient microorganisms, which eventually died from oxygen poisoning.

Modern cyanobacteria continue to exert disproportionate influence for their size. The Prochlorococcus group of cyanobacteria—which measure in at less than a micron in diameter, allowing 500-plus individuals to fit comfortably on the head of a pin—account for a significant fraction of global photosynthesis by virtue of their ubiquitous presence in nutrient-depleted ocean waters. Even tinier agents—the viruses that infect these bacteria, called cyanophages—appear capable of wielding equally surprising influence on global cycles by affecting the population dynamics and evolutionary path of Prochlorococcus.

To better understand the nature of virus–host interactions at sea, Sallie Chisholm and colleagues investigated the genetic makeup of three cyanophages. The marine phages resemble two terrestrial phages—called T4 and T7—that infect Escherichia coli but carry genes that appear specially adapted to infecting photosynthetic bacteria in nutrient-poor oceans.

Of over 430 completed phage genomes, only one (P60) infects cyanobacteria. Since marine phages likely face different selection pressures than their terrestrial equivalents, the authors explain, genome analysis can shed light on the agents of selection, besides providing a survey of marine phage types. Chisholm and colleagues chose to sequence three marine phages—one podovirus (P-SSP7) and two myoviruses (P-SSM2 and P-SSM4)—based on their morphology and host range, and characterized their genomes. The P-SSP7 virus has genes that closely match many of T7's so-called core genes—signature genes required for that virus's mode of infection, which involves killing its host. P-SSP7 also has the same genome structure as other T7-like phages, though it appears capable of coexisting with its host (based on the presence of an integrating enzyme) while T7 kills as it infects. Chisholm and colleagues go on to characterize the two myoviruses and find that both viruses share most of the core genes found in T4-like phages. And like T4 phages, both myoviruses lack the integrating enzymes, suggesting they share T4 phages' homicidal approach to infection.[Fig pbio-0030184-g001]

**Figure pbio-0030184-g001:**
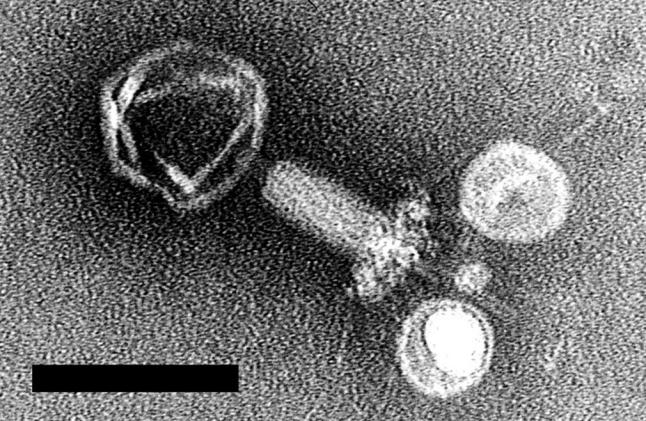
Despite a striking resemblance to an E. coli virus, this marine virus appears to have evolved genes adapted to infecting photosynthetic bacteria inhabiting low-nutrient oceans (Scale bar indicates 100 nm) (Photo: Peter Weigele)

Beyond the core phage genes, Chisholm and colleagues also present a survey of genes likely derived from cyanobacteria that “could play defining functional roles” in marine phage–host interactions. All three cyanophages contain photosynthesis-related genes, some of which, the authors propose, may mean the virus helps the host maintain photosynthesis during infection. The podovirus also has a candidate gene involved in DNA synthesis, which the authors speculate might help the virus reproduce in nutrient-poor environments, and all three cyanophages carry genes involved in metabolizing carbon. The absence of such genes in terrestrial phages, the authors argue, lends support to the notion that marine phages have evolved different adaptive mechanisms in response to the ocean environment.

Given the intimate relation between virus and host, the effects of gene swapping between virus and host is likely to be a two-way street. Just as cyanophages may help shape the fate of their hosts, it's likely that cyanobacterial genes influence phage ecology and perhaps even its range. The cyanophages characterized here take after two phages that were central to many fundamental breakthroughs in molecular biology, including the discovery that genes are made of DNA. It remains to be seen how the marine versions of these legendary laboratory viruses contribute to our understanding of phage infections in one of the most abundant, ecologically diverse primary producers in the open seas. See also the related Primer “The Third Age of Phage” (DOI: 10.1371/journal.pbio.0030182).

